# Influence of extrusion cooking on physicochemical properties and starch digestion kinetics of *Sphenostylis stenocarpa*, *Cajanus cajan*, and *Vigna subterranean* grains

**DOI:** 10.1371/journal.pone.0242697

**Published:** 2020-12-01

**Authors:** Oluwafunmilayo O. Adeleye, Seun T. Awodiran, Atinuke O. Ajayi, Toluwalope F. Ogunmoyela

**Affiliations:** Agricultural Biochemistry and Nutrition Laboratory, Department of Animal Science, University of Ibadan, Ibadan, Nigeria; Indian Institute of Food Processing Technology (IIFPT), INDIA

## Abstract

Thermal degradation of sugars and amino acids, and depolymerization of macromolecules such as starch, proteins and fibre occasioned by high-temperature short-time extrusion cooking modify the physicochemical and functional properties of raw materials. High-temperature short-time extrusion cooking holds promise for the expanded use of non-conventional ingredients as food/feed due to its practicality, increased productivity and efficiency, and ability to retain thermally degradable nutrients during cooking. However, little is known about the effect of the high-temperature short-time extrusion cooking process on the physicochemical properties and starch digestibility of lesser-known grain legumes such as African yam beans (*Sphenostylis stenocarpa*), Pigeon pea (*Cajanus cajan*), and Bambara peanut (*Vigna subterranean*). In this study, we investigate the effect of high-temperature short-time extrusion cooking and extrusion cooking temperature; low (100°C) vs high (140°C) temperatures in a single screw extruder, on hydration characteristics, viscoamylolytic properties, *in vitro* starch digestibility and digestion kinetics of these grain legumes. We show that water holding capacity and swelling power increased (p < 0.05) with increasing extrusion temperature for *Sphenostylis stenocarpa* and *Vigna subterranean* but not *Cajanus cajan* extrudates. Significant effects of extrusion cooking (i.e unextruded *vs* 100°C and unextruded *vs* 140°C) and extrusion temperatures (i.e. 100°C *vs* 140°C) were observed in peak, trough, final and setback viscosities of all extrudates. Starch digestibility and digestion characteristics were modified with increase in extrusion temperature, however, no effect of extrusion temperatures (i.e. 100°C *vs* 140°C) on starch digestion kinetics was observed for *Sphenostylis stenocarpa* and *Vigna subterranean* except for hydrolysis index (34.77 *vs* 40.77%). Nutritional and physiological implications of extruded grain legumes in monogastric animal feeding were also highlighted. The Information presented herein will influence expanded use of extruded grain legumes as feed ingredients for intensive monogastric animal feeding.

## Introduction

Legume grains are protein-energy rich seeds, members of the dicotyledonous family *Fabaceae* or *Leguminosae* commonly found in the tropical rain forests of the Americas and Africa [1]. Grain legumes have an 11–50% protein content [2], hence the emphasis on grain legumes as dietary sources of protein, especially among low-income populations. The energy yield of grain legumes range from 905–1804 kJ/100g [3] and complex carbohydrate constituents range from 65–72% on dry matter basis. Starch is the most abundant complex carbohydrate fraction (22–45% of legume grains), followed by non-starch polysaccharides ((NSPs) also loosely translated as dietary fibre) representing 10–20% of complex carbohydrates and the balance being sugars [4,5]. Oligosaccharides, polysaccharides (e.g. cellulose, hemicellulose, pectic polysaccharides, and resistant starch) and associated plant substances[6], constitute the NSPs of grain legumes. Oligosaccharides of grain legumes are hailed for their prebiotic activity i.e. their ability to selectively stimulate the proliferation of one or more beneficial bacterial species in the hindgut, as they confer benefits to colonic health to a greater degree than resistant starch [7]. Sucrose galactosides; raffinose, stachyose and verbascose, represent the most researched oligosaccharides and account for 31–76% of total sugars in grain legumes. Carbohydrates of grain legume origin are often termed “low glycaemic” as their consumption produce a smaller rise in blood glucose relative to a reference, often white bread or pure glucose, owing to their high soluble and insoluble fibre, and resistant starch content which range from 5–35% by weight [8,9]. The glycaemic index of foods is influenced by a range of factors which include their botanical origin, varietal differences, type of starch, physical structure of the carbohydrate (e.g. particle size, granular dimensions and presence of associated compounds in the food/feed matrix e.g. lipids, proteins, fibres and acids), extent of processing and storage, and degree of gelatinization of inherent starch. Glycaemic index ranges from 0–100, with grain legumes having a glycaemic index of ≦ 55 and pure glucose = 100 [9–13].

Grain legumes also provide dietary sources of antioxidants such as polyphenols, flavonoids, isoflavones and anthocyanins, which protect body cells against free radicals by functioning as radical scavengers, reducing agents, potential complexes of pro-oxidant metals and quenchers of singlet oxygen [14]. Other bioactive compounds of grain legumes are enzyme inhibitors (e.g. serine protease inhibitors and amylase inhibitors), phytic acid, haemagglutinins, condensed tannins, and lectins, which possess antinutritional properties. The presence of these antinutritional bioactive compounds in grain legumes impair their extensive use in foods and feeds. However, when grain legumes are subjected to dry or hydrothermal heat processes, heat labile bioactives are deactivated, alienating the antinutritional properties attached to them [15–17].

In high-temperature short-time (HTST) extrusion cooking, a moisture, temperature, pressure and mechanical shear regime is applied to the grains/ingredients [18]. Compared with other hydrothermal/thermal processes such as roasting, boiling, microwaving, and autoclaving, extrusion cooking affords a continuous consolidated multi-operational (involving mixing, heating, pressure cooking and mechanical shearing) process which is energy efficient and produces a high-quality output [19]. Adoption of HTST extrusion by the food and feed industry has resulted in increased production of ready-to-eat snacks and cereals, precooked infant meals, meat and cheese analogues, dry pet foods, fish and livestock ingredients and concentrates using lesser known cereals and legumes [20–27]. This heat processing technique also modifies their molecular structures and nutritional profile. Notable modifications to carbohydrates of grain legumes accruing from HTST extrusion cooking influence their hydration and functional properties, enzyme susceptibility[28], glycaemic index/potential and starch digestibility [29]. Studies investigating the influence of HTST extrusion cooking on enzyme susceptibility in grain legumes have reported an increase of 32.05%, 43.70% and 58.27% in starch digestibility of Lentils (*Lens culinaris Medikus*) in twin screw extruders at 140, 160 and 180°C respectively [30] and 21–22% increased enzyme susceptibility in single screw extruded *Phaseolus vulgaris* L. at 150°C [31]. Increase in enzyme susceptibility of materials after HTST extrusion cooking is attributed to macromolecular degradation of their starch granules [32] i.e. reduction in relative molecular weight of starch polysaccharide chains resulting from shearing of glycosidic bonds in amylopectin and amylose chains during the cooking process [32,33]. The combination of high temperature and moisture employed in extrusion cooking, also disrupts intragranular hydrogen bonds allowing for association of water with free hydroxyl groups and resulting in modified functional and rheological properties [34].

These physical, chemical and nutritional changes in foods and feeds occasioned by HTST extrusion cooking, hold implications for humans and monogastric (single-chambered stomach) animals who consume the bulk of extruded products. The monogastric gastrointestinal tract is involved in mechanical digestion (chewing or mechanical pulverization of food materials in the avian gizzard) and enzymatic digestion, moving digested food thorough the body, and absorbing the products of digestion. The process of extrusion cooking is known to considerably impact nutrient availability[35], palatability, rate and extent of digestion of carbohydrates [36] in monogastric animals, while destroying enzyme inhibitors and toxins, reducing wastage as well as improving food/feed shelf-life and hygiene [35,37].

Lesser known grain legumes such as *Sphenostylis stenocarpa*, *Cajanus cajan*, and *Vigna subterranean* have attracted little research into their response to HTST extrusion cooking, hence limiting their use in foods and feeds even within geographical locations that boast of local production. This study describes the influence of high-temperature short-time extrusion cooking temperatures on the hydration and viscoamylolytic properties of *Sphenostylis stenocarpa*, *Cajanus cajan*, and *Vigna subterranean*, and their susceptibility to enzymatic hydrolysis in a monogastric digestive system model. It further postulates nutritional and physiological implications of these modifications in monogastric animals.

## Materials and methods

### Legume flour preparation and extrusion cooking

*Sphenostylis stenocarpa* (African yam beans), *Vigna subterranean* (Bambara groundnut) and *Cajanus cajan* (Pigeon pea), were sourced from a local grain supplier, cleaned and milled through a 1mm sieve mesh. Moisture was adjusted to 25g/100g sample and mixed manually before subjecting to high-temperature short-time extrusion cooking in a single screw laboratory-scale extruder. Extruder characteristics were: screw diameter– 18.5mm, screw length– 304mm, screw speed—60 rpm, and temperature measured at the outlet die -100°C (low temperature treatment) and 140°C (high temperature treatment), respectively. The choice of extrusion conditions; moisture and extrusion temperatures was based on earlier studies [15,38]. After extrusion, samples were dried in a forced air oven at 50°C overnight [38] and subsequently milled and stored in polyethylene bags at 4°C.

### Hydration properties

Water retention capacity (WRC) was determined by methods outlined by AACC [39], with slight modifications. Briefly, 10% (w/v) of unextruded and extruded samples was prepared in pre-weighed tubes, vigorously vortexed for 1 min, held at room temperature for 30 mins and subsequently centrifuged at 1590 × g for 15 minutes. The unabsorbed liquid was decanted, absorbed water was calculated by difference and presented as a percentage of the dry sample weight. Water solubility index (WSI) and swelling power (SP) were determined at 70°C by the methods of Leach et al., [40] with slight modifications. Briefly, 10% (w/v) of unextruded and extruded samples was prepared, stirred for 30 minutes at 70°C and subsequently centrifuged at 7000 × g for 20 minutes. Supernatant was aspirated into a pre-weighed crucible and dried at 105°C for 24 hours, and the dry solids weighed at the end of the drying period. The swelling power of the samples was calculated as the ratio of the weight of moisture imbibed by the granules of the sample to the weight of the dry samples while solubility index was calculated as the ratio of the weight of soluble solids to the weight of the dry sample and presented as a percentage.

### Viscoamylolytic properties

Viscoamylolytic indices; peak, trough, breakdown, setback and final viscosities, time at peak viscosity and pasting temperature—were determined in a Rapid Visco TM Analyzer RVA-4 (Newport Scientific Pty. Ltd., NSW, Australia) with the software program Thermocline for Windows according to AACC method 61-02-01 [39]. About 2g of unextruded or extruded legume grain flour (corrected to 14% moisture) and 25 mL distilled water were combined and stirred at a paddle speed of 960 rpm for the first 10 seconds then reduced to 160 rpm. Pasting profile adopted involved holding temperature at 50°C for 1 minute, raising to 95°C in 3.75 minutes and holding for 2.5 minutes, then cooling to 50°C in 3.75 minutes and holding for a final 5 minutes.

### Enzyme susceptibility test and starch digestion kinetics

Digestion was simulated under total tract conditions consisting of a three step hydrolysis mimicking digestion in the mouth, stomach and small intestine within a closed system according to the method of Sopade and Gidley [41], and glucose concentration measured by glucometry (ACCUCHEK active, Roche Diagnostics GmbH, Mammheim, Germany) after incubation at 0, 15, 30, 60, 90, 120, 180 and 240 mins. Zero-hour was defined as the beginning of the small intestine simulation step, as this connotes the main site of starch hydrolysis in monogastric animals [38]. Glucose concentrations obtained were converted to starch by a correction factor of 0.9. Starch digestion kinetics was studied by fitting time point glucose concentration data to a first-order exponential model [42] using the SOLVER add-in function in EXCEL [43]. Area under the digestogram (AUC) and hydrolysis index (HI) for each flour was computed up to 180 mins of incubation, using white bread as a reference product. Predicted glycaemic index (pGI) was also calculated for each flour [44].

### Calculations and statistical analysis

All determinations were conducted in quadruplicate except the RVA determination which was conducted in duplicate. Data was subjected to one way analysis of variance (ANOVA), contrasts to assess the effects of extrusion (unextruded *vs* 100°C extrudates and unextruded *vs* 140°C extrudates) and extrusion temperatures (100°C extrudates *vs* 140°C extrudates) were done using non-orthogonal contrast (SPSS version 20), and means separated at P<0.05.

## Results

Proximate composition of the raw and extruded *Sphenostylis stenocarpa* (African yam beans), *Vigna subterranean* (Bambara groundnut) and *Cajanus cajan* (Pigeon pea) products are shown in Table 1, and have been discussed in detail in a previous publication [15].

**Table 1:** Proximate composition of raw and extruded *Sphenostylis stenocarpa* (African yam beans), *Vigna subterranean* (Bambara groundnut) and *Cajanus cajan* (Pigeon pea) flours^†^.

		Crude protein (%)	Crude fibre (%)	Ether extract (%)	Nitrogen-free extract %)	Ash (%)
African yam beans	AYR	20.54 ± 0.05	4.38 ± 0.20	10.25 ± 1.08	52.52 ± 0.95	2.95 ± 0.00
	AY100	20.21± 0.02	4.83 ± 0.15	4.62 ± 2.04	58.74 ± 2.07	2.90 ± 0.05
	AY140	18.22 ± 0.01	4.28 ± 0.30	2.15 ± 0.50	63.53 ± 0.17	3.18 ± 0.13
Bambara groundnut (*Vigna subterrenean*)	BBR	20.50 ± 0.01	4.03 ± 0.05	11.45 ± 1.28	51.42 ± 1.27	2.90 ± 0.00
	BB100	20.27 ± 0.01	6.13 ± 0.46	5.15 ± 0.65	55.87 ± 0.87	3.63 ± 0.08
	BB140	19.91 ± 0.01	6.03 ± 0.06	0.77 ± 0.18	60.36 ± 0.16	3.63 ± 0.03
Pigeon pea (*Cajanus cajan*)	PPR	21.20 ± 0.02	7.72 ± 0.25	8.50 ± 3.10	48.59 ± 2.74	4.88 ± 0.03
	PP100	20.07 ± 0.00	8.02 ± 0.02	3.37 ± 0.16	55.58 ± 0.61	4.50 ± 0.20
	PP140	18.66 ± 0.01	9.35 ± 0.13	2.03 ± 0.81	55.60 ± 0.69	5.02 ± 0.12

Values are presented as means of triplicate determinations ± standard deviation.

AYR—unextruded African yam beans (*Sphenostylis stenocarpa*); AY100—African yam beans (*Sphenostylis stenocarpa*) extruded at 100°C; AY140—African yam beans (*Sphenostylis stenocarpa*) extruded at 140°C; BBR—unextruded Bambara groundnut (*Vigna subterranean*); BB100—Bambara groundnut (*Vigna subterranean*) extruded at 100°C; BB140—Bambara groundnut (*Vigna subterranean*) extruded at 140°C; PPR—unextruded Pigeon pea (*Cajanus cajan*); PP100—Pigeon pea (*Cajanus cajan*) extruded at 100°C; PP140—Pigeon pea (*Cajanus cajan*) extruded at 140°C.

^†^[15].

### Hydration properties

**Fig 1:**
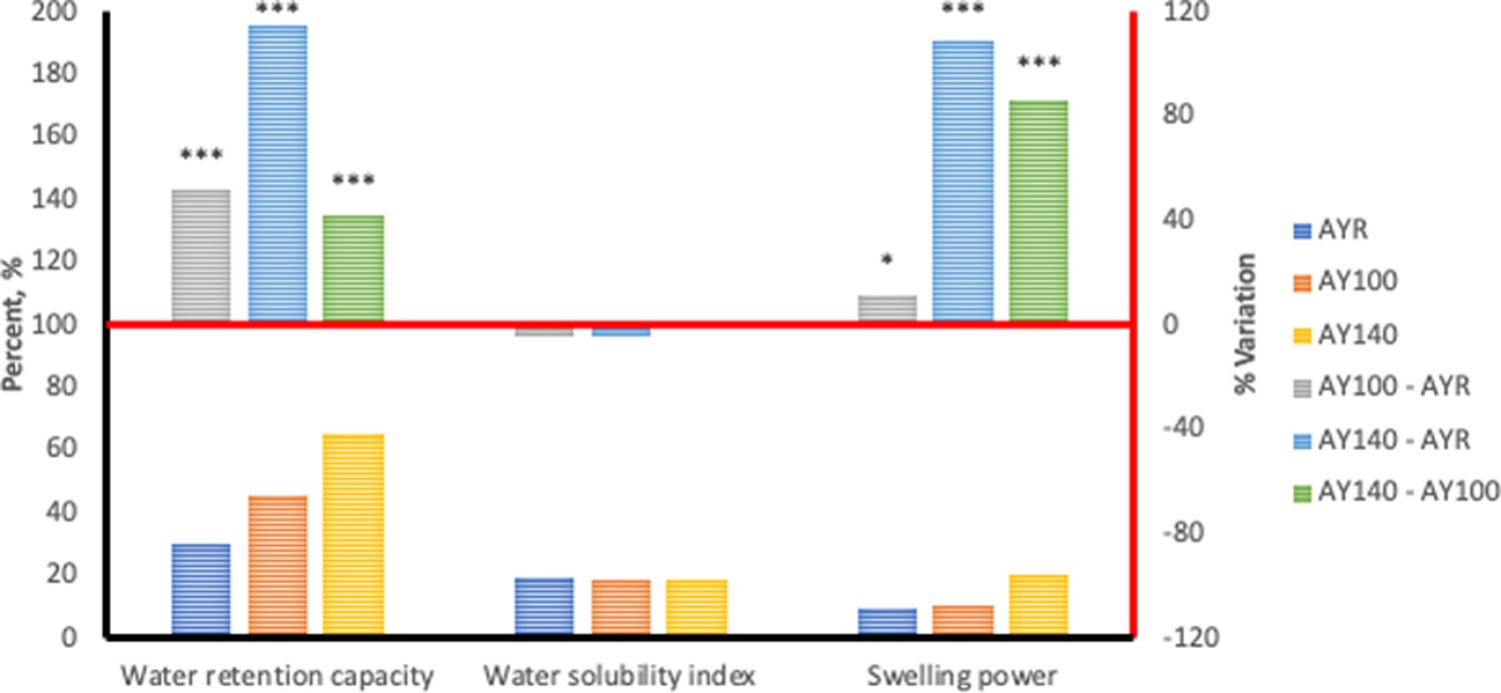
Hydration properties associated with HTST extrusion of African yam beans (*Sphenostylis stenocarpa*) at different temperatures. AYR—unextruded African yam beans (*Sphenostylis stenocarpa*); AY100—African yam beans (*Sphenostylis stenocarpa*) extruded at 100°C; AY140—African yam beans (*Sphenostylis stenocarpa*) extruded at 140°C.

**Fig 2:**
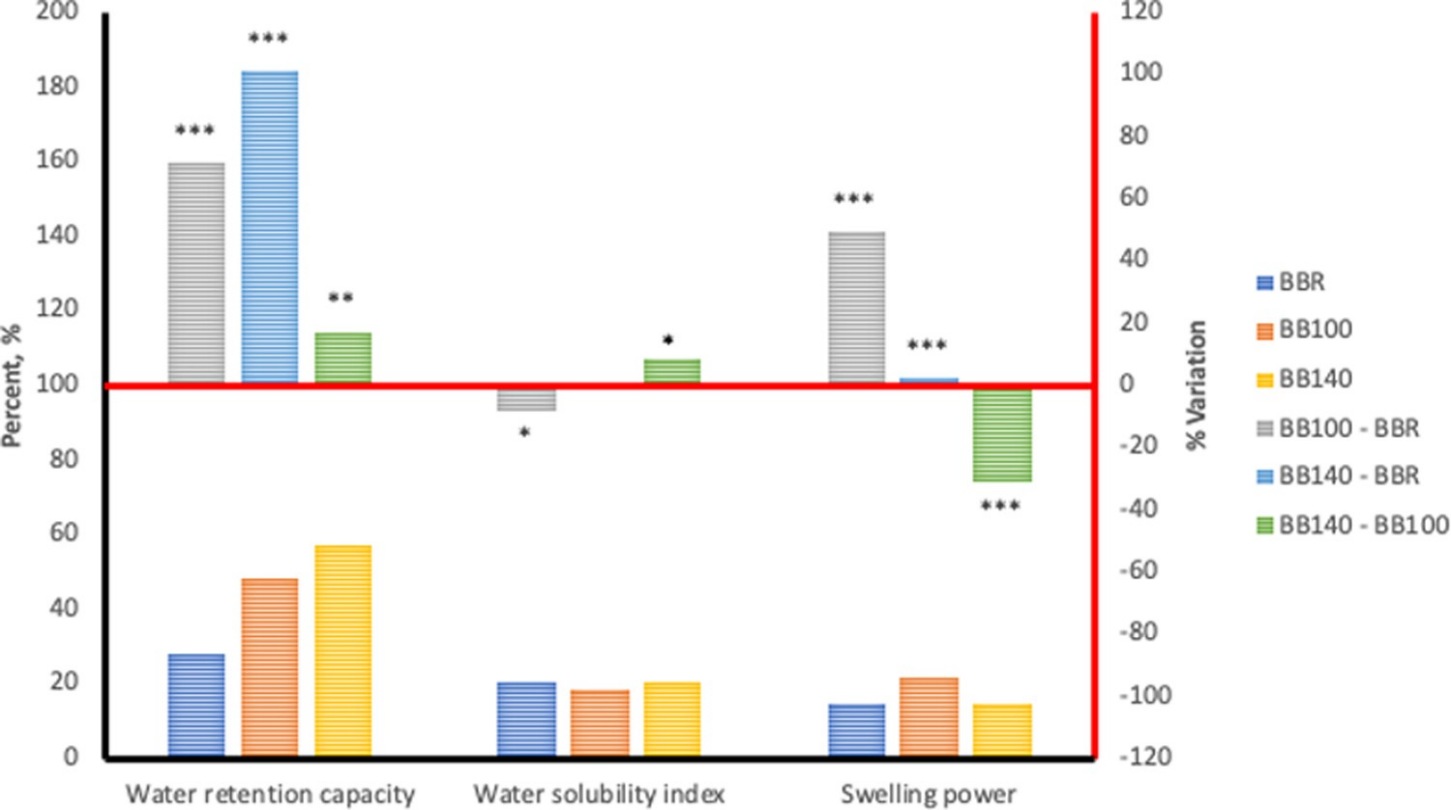
Hydration properties associated with HTST extrusion of Bambara groundnut (*Vigna subterranean*) at different temperatures. BBR—unextruded Bambara groundnut (*Vigna subterranean*); BB100—Bambara groundnut (*Vigna subterranean*) extruded at 100°C; BB140—Bambara groundnut (*Vigna subterranean*) extruded at 140°C.

**Fig 3:**
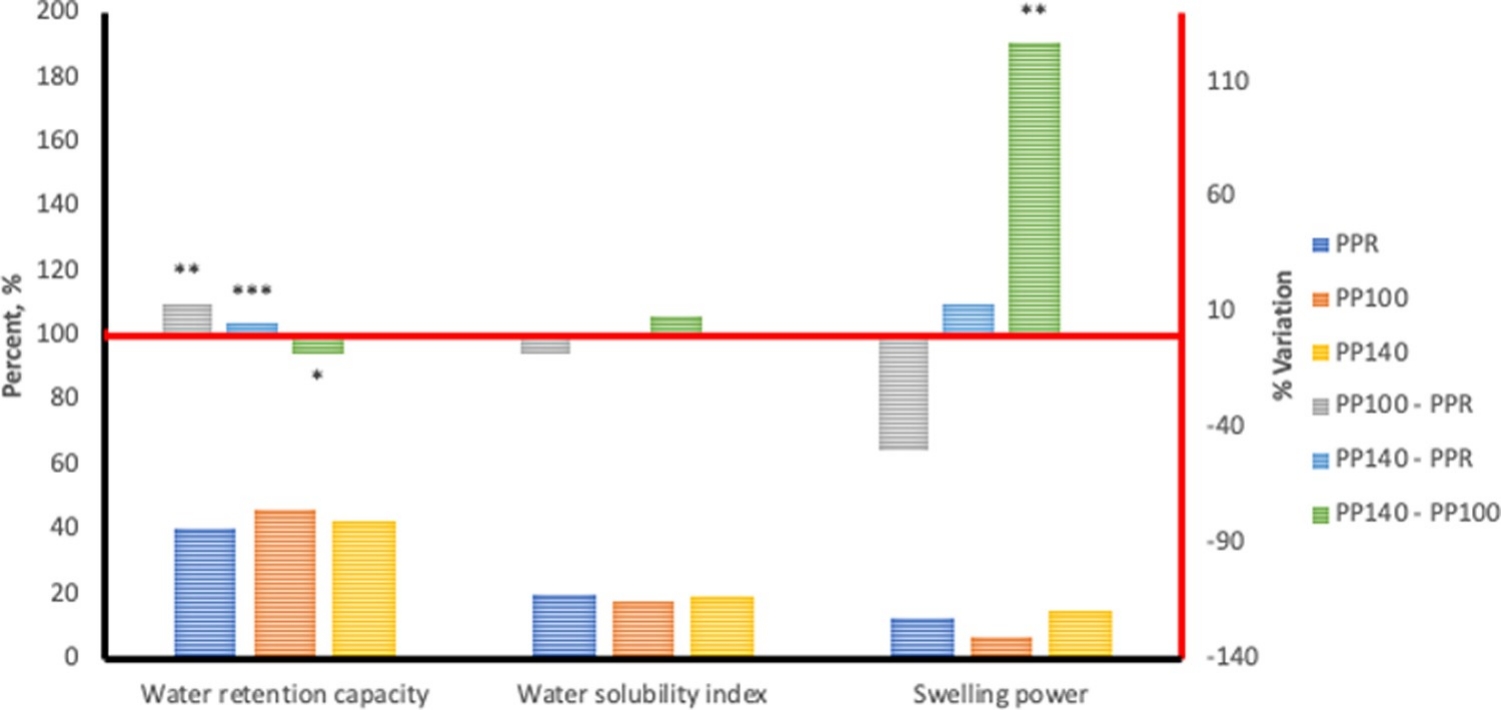
Hydration properties associated with HTST extrusion of Pigeon pea (*Cajanus cajan*) at different temperatures. PPR—unextruded Pigeon pea (*Cajanus cajan*); PP100—Pigeon pea (*Cajanus cajan*) extruded at 100°C; PP140—Pigeon pea (*Cajanus cajan*) extruded at 140°C.

Hydration properties of any plant-based material provides an evaluation of the water affinity of their functional polymeric components which is primarily starch. The hydration capacities of unextruded and extruded flours of *Sphenostylis stenocarpa*, *Cajanus cajan*, and *Vigna subterranean* are presented in bar charts in Figs 1–3.

Water retention capacity (WRC) measures the ability of food/feed ingredients to bind, imbibe and retain water within their matrix. Extrusion cooking at 100°C and 140°C significantly increased WRC by 52.69% and116.6% respectively, in *Sphenostylis stenocarpa*, 75.73% and 105.9% respectively, in *Vigna subterranean* and 13.98% and 5.08% respectively, in *Cajanus cajan*. Increase in extrusion cooking temperature from 100°C to 140°C also significantly increased WRC by 41.71% in *Sphenostylis stenocarpa*, and17.21% in *Vigna subterranean*, whilst decreasing WRC by 7.64% in *Cajanus cajan*. Swelling power measures granular swelling accruing to intergranular and intragranular imbibition of water, and was measured at 70°C in the current study. On the other hand, solubility index indicates the extent of “melting” occurring within the crystalline regions of starch. Swelling power in *Sphenostylis stenocarpa* extrudates increased with increased extrusion temperature and ranged from 11.93–108.9% over the unextruded *Sphenostylis stenocarpa*, with an 87.73% higher SP recorded in 140°C extrudates compared to 100°C extrudates of *Sphenostylis stenocarpa*. A significantly higher increase in SP was observed at 100°C extrusion than at 140°C i.e. 72.19% and 157.3% respectively, over SP of the unextruded *Vigna subterranean* flour, and a 49.36% decline in SP due to extrusion at the high temperature (BB100 *vs* BB140; p = 0.001). In contrast, No effect of extrusion cooking at 100°C or 140°C was observed on SP (PPR *vs* PP100 = 0.09 and PPR *vs* PP140 = 0.65), however, a 143.4% higher SP was recorded for when *Cajanus cajan* was extruded at 140°C compared to 100°C extrudates (PP100 *vs* PP140; p = 0.04). Extrusion cooking or extrusion cooking temperatures did not affect WSI of *Sphenostylis stenocarpa* and *Cajanus cajan* products. However a marginal reduction (-5.11%) in response to extrusion cooking at 100°C (BBR *vs* BB100; p = 0.03) as well as a marginal increase of +4.84% in response to extrusion cooking temperatures (BB100 *vs* BB140; p = 0.03) were observed, with similar WSI observed in BBR and BB140 (p > 0.05).

### Viscoamylolytic properties

The effect of HTST extrusion cooking temperatures on viscoamylolytic properties of unextruded and extruded flours of *Sphenostylis stenocarpa*, *Cajanus cajan*, and *Vigna subterranean* is presented in Table 2.

**Table 2:** Effect of high-temperature short-time extrusion cooking temperatures on viscoamylolytic indices of *Sphenostylis stenocarpa* (African yam beans), *Vigna subterranean* (Bambara groundnut) and *Cajanus cajan* (Pigeon pea) flours.

	Viscoamylolytic indices (cP)						
	Peak viscosity	Trough viscosity	Breakdown viscosity	Final viscosity	Setback viscosity	Pasting temperature (°C)	Peak time (mins)
AYR	1029.5 ± 33.23	909.0 ± 57.98	120.5 ± 24.75	1282.5 ± 61.52	373.5 ± 3.54	82.3 ± 0.07	4.9 ± 0.19
AY100	100.5 ± 4.95	81.5 ± 3.54	19.0 ± 1.41	187.5 ± 9.19	106.0 ± 5.66	68.0 ± 2.83	6.9 ± 0.09
AY140	189.0 ± 38.18	163.0 ± 22.63	26.0 ± 15.56	282.5 ± 6.36	119.5 ± 16.26	55.8 ± 7.35	7.0 ± 0.00
*Contrast P-values*							
AYR vs AY100	*	*	NS	*	***	NS	*
AYR vs AY140	**	*	NS	*	*	0.01	*
AY100 vs AY140	NS	NS	NS	NS	NS	NS	NS
PPR	560 ± 8.49	546 ± 12.73	14.0 ± 4.24	760.0 ± 18.39	214.0 ± 5.67	86.73 ± 0.57	6.8 ± 0.28
PP100	42.0 ± 12.73	32.5 ± 7.78	9.5 ± 4.95	71.0 ± 11.31	38.5 ± 3.54	81.00 ± 0.00	7.0 ± 0.00
PP140	62.5 ± 4.95	53.0 ± 4.24	9.5 ± 0.71	93.5 ± 7.78	40.50 ± 3.54	nd	7.0 ± 0.00
*Contrast P-values*							
PPR vs PP100	***	***	NS	***	**	***	NS
PPR vs PP140	***	**	NS	**	**		NS
PP100 vs PP140	NS	NS	NS	NS	NS		NS
BBR	818.0 ± 42.43	791.5 ± 47.38	26.5 ± 4.95	1826.5 ± 70.00	1035 ± 22.63	85.2 ± 0.57	6.8 ± 0.00
BB100	134.5 ± 24.75	109.0 ± 19.80	25.5 ± 4.95	213.5 ± 20.51	104.5 ± 0.71	74.58 ± 3.47	7.0 ± 0.00
BB140	148.5 ± 19.09	126.0 ± 15.56	22.5 ± 3.54	249.0 ± 41.01	123.0 ± 25.46	70.58 ± 0.60	6.9 ± 0.09
*Contrast P-values*							
BBR vs BB100	**	*	NS	*	*	**	NS
BBR vs BB140	*	*	NS	**	***	**	NS
BB100 vs BB140	NS	NS	NS	NS	NS	NS	NS

Values are presented as means ± standard deviation.

*** P ≤ 0.001

** P ≤ 0.01

* P < 0.05 NS–not significant, P > 0.05.

nd—not determined.

Significant decline in peak, trough, final and setback viscosities in response to extrusion cooking temperatures (i.e. unextruded vs 100°C and unextruded vs 140°C) was recorded for all grain legumes investigated, however, no significant effect (p > 0.05) of extrusion cooking temperatures was observed for breakdown viscosity. No significant effect (p > 0.05) of extrusion temperatures (i.e. 100°C vs 140°C) was also observed for all viscoamylolytic indices (i.e. peak, trough, breakdown, final and setback viscosities, pasting temperature and time at peak viscosity), measured in the current study. However, pasting temperature was significantly reduced (p = 0.01) in 140°C extrudates of *Sphenostylis stenocarpa*, and 100°C extrudates of *Cajanus cajan* (p = 0.001). Extrusion of *Vigna subterranean* flours at both 100°C and 140°C significantly lowered pasting temperature (p = 0.01). Time at peak viscosity was significantly increased by extrusion at either 100°C or 140°C in *Sphenostylis stenocarpa* extrudates only.

### Starch digestion kinetics and *in vitro* starch digestibility

**Fig 4:**
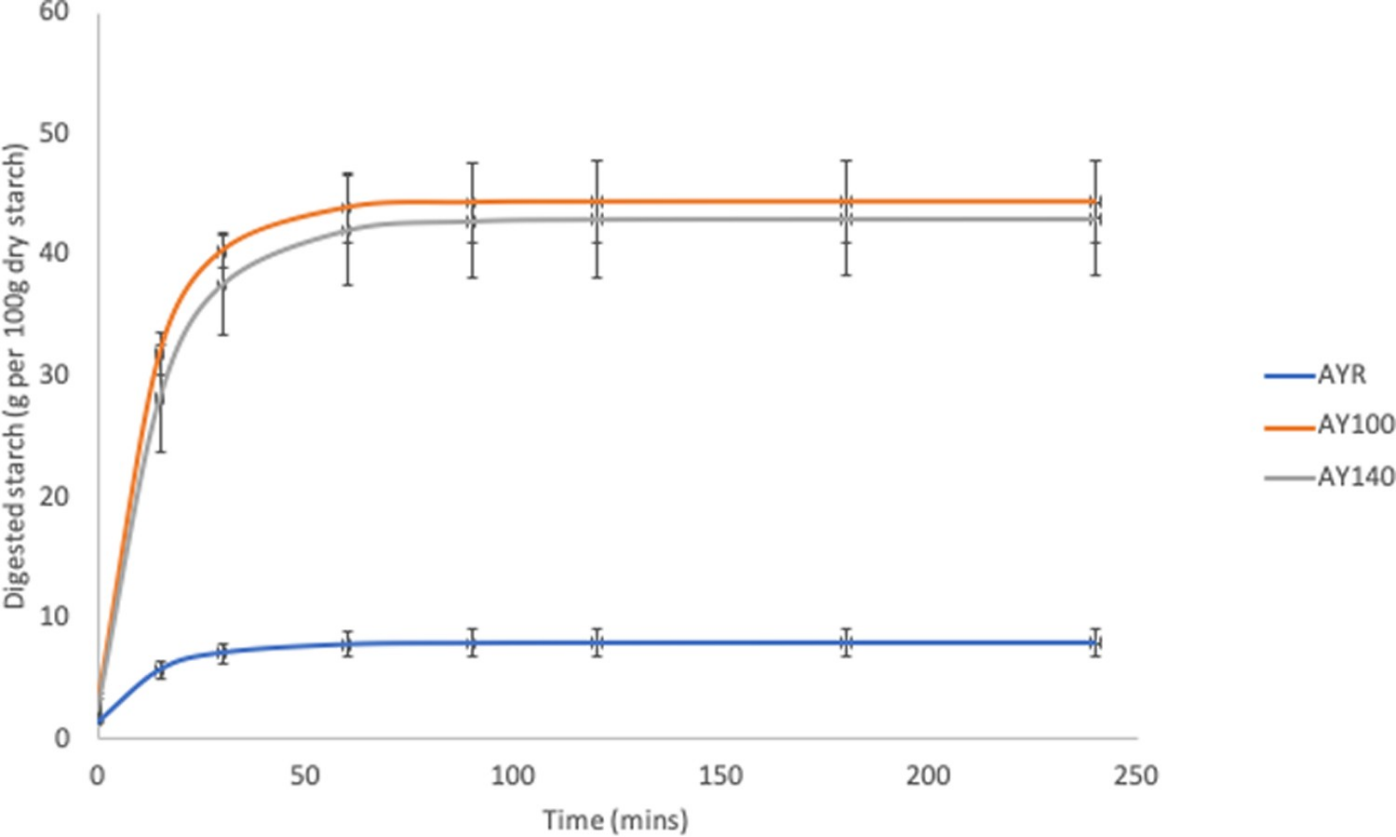
Digestion pattern associated with HTST extrusion of African yam beans (*Sphenostylis stenocarpa*) at different temperatures. AYR—unextruded African yam beans (*Sphenostylis stenocarpa*); AY100—African yam beans (*Sphenostylis stenocarpa*) extruded at 100°C; AY140—African yam beans (*Sphenostylis stenocarpa*) extruded at 140°C.

**Fig 5:**
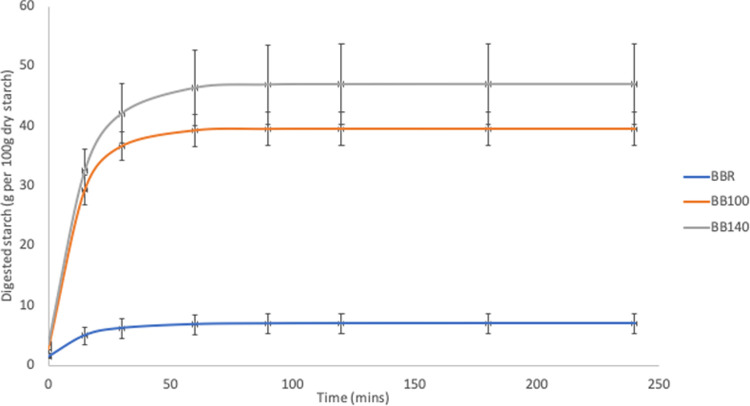
Digestion pattern associated with HTST extrusion of Bambara groundnut (*Vigna subterranean*) at different temperatures. BBR—unextruded Bambara groundnut (*Vigna subterranean*); BB100—Bambara groundnut (*Vigna subterranean*) extruded at 100°C; BB140—Bambara groundnut (*Vigna subterranean*) extruded at 140°C.

**Fig 6:**
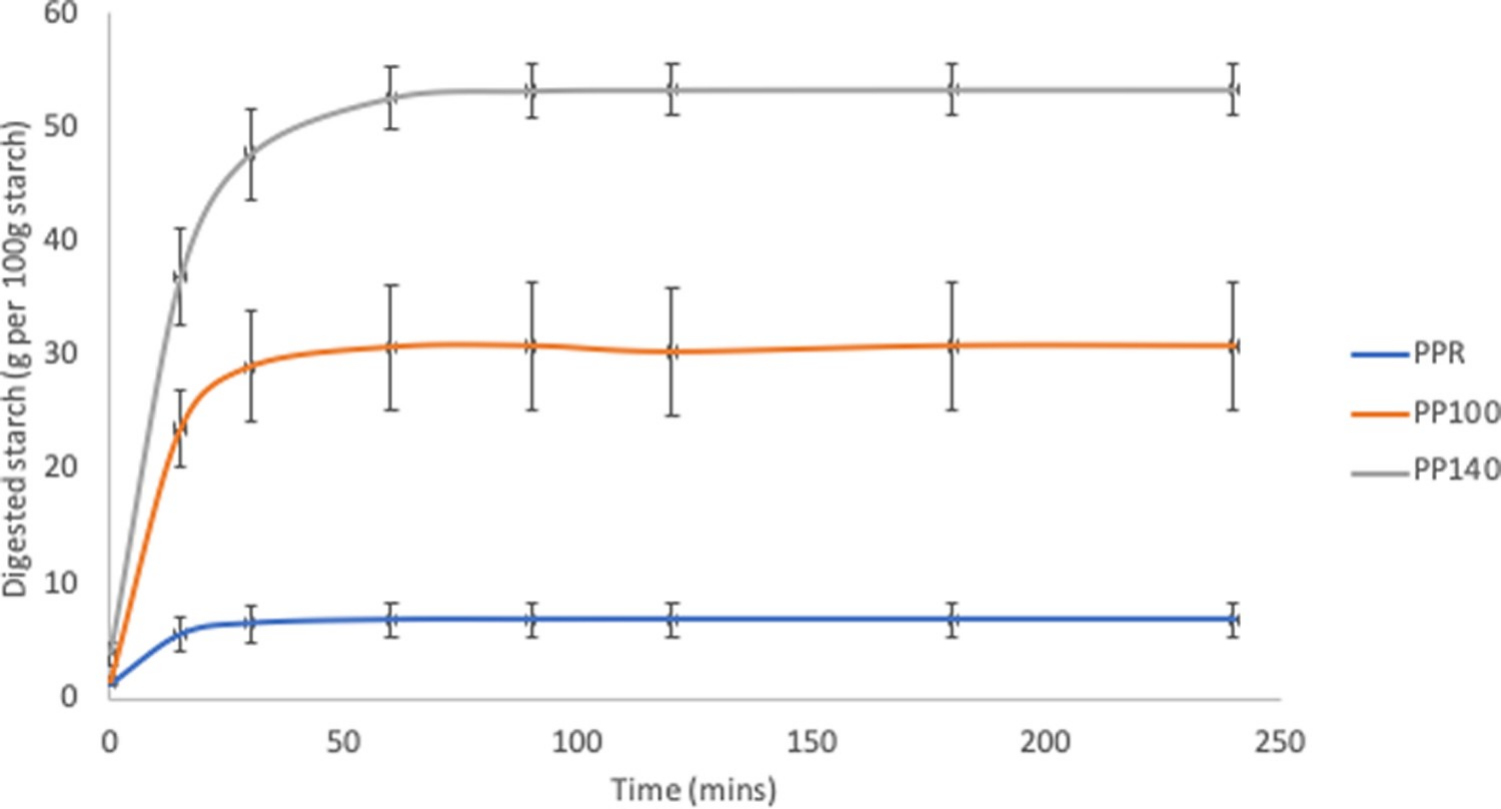
Digestion pattern associated with HTST extrusion of Pigeon pea (*Cajanus cajan*) at different temperatures. PPR—unextruded Pigeon pea (*Cajanus cajan*); PP100—Pigeon pea (*Cajanus cajan*) extruded at 100°C; PP140—Pigeon pea (*Cajanus cajan*) extruded at 140°C.

Table 3 and Figs 4–6 show the *in vitro* starch digestion pattern and starch digestion kinetics associated with HTST extrusion cooking of *Sphenostylis stenocarpa*, *Cajanus cajan*, and *Vigna subterranean* at different temperatures.

**Table 3:** Effect of high-temperature short-time extrusion cooking temperatures on starch digestion kinetics of *Sphenostylis stenocarpa* (African yam beans), *Vigna subterranean* (Bambara groundnut) and *Cajanus cajan* (Pigeon pea) flours.

	D_0_	K × 10^−3^	AUC × 10^3^	HI	pGI
AYR	1.44 ± 0.14	75.44 ± 18.89	1.32 ± 0.17	6.94 ± 0.79	44.81 ± 0.49
AY100	2.82 ± 0.85	83.27 ± 21.47	7.39 ± 0.46	38.89 ± 3.72	70.47 ± 2.79
AY140	2.43 ± 0.96	75.16 ± 29.87	7.07 ± 0.73	37.25 ± 5.00	68.73 ± 3.68
*Contrast P-values*					
AYR vs AY100	*	NS	***	***	***
AYR vs AY140	NS	NS	***	***	***
AY100 vs AY140	NS	NS	NS	NS	NS
PPR	1.37 ± 0.31	98.55 ± 26.94	1.17 ± 0.27	6.16 ± 1.54	44.28 ± 1.27
PP100	1.61 ± 0.53	94.72 ± 9.25	5.18 ± 0.91	27.10 ± 3.99	61.25 ± 3.29
PP140	3.90 ± 0.97	73.78 ± 12.95	8.83 ± 0.46	46.42 ± 3.11	76.34 ± 3.01
*Contrast P-values*					
PPR vs PP100	NS	NS	***	***	***
PPR vs PP140	***	NS	***	***	***
PP100 vs PP140	***	*	***	***	***
BBR	1.37 ± 0.16	71.26 ± 27.15	1.16 ± 0.29	6.06 ± 1.45	44.07 ± 1.14
BB100	2.67 ± 0.68	87.42 ± 15.12	6.60 ± 0.45	34.77 ± 3.62	67.34 ± 2.87
BB140	3.55 ± 1.01	75.19 ± 11.46	7.78 ± 1.06	40.77 ± 4.52	71.86 ± 3.67
*Contrast P-values*					
BBR vs BB100	*	NS	***	***	***
BBR vs BB140	*	NS	***	***	***
BB100 vs BB140	NS	NS	NS	***	NS

Values are presented as means ± standard deviation.

*** P ≤ 0.001

** P ≤ 0.01

* P < 0.05 NS–not significant, P > 0.05.

D_o_—Digested starch at time, t = 0 (equivalent to very rapidly digesting starch [42], g/100g dry starch); K—rate constant (min^-1^); AUC—area under the digestogram (mg dL^-1^ · 240 min^-1^); HI -hydrolysis index; pGI -predicted glycemic index [44].

**Fig 7:**
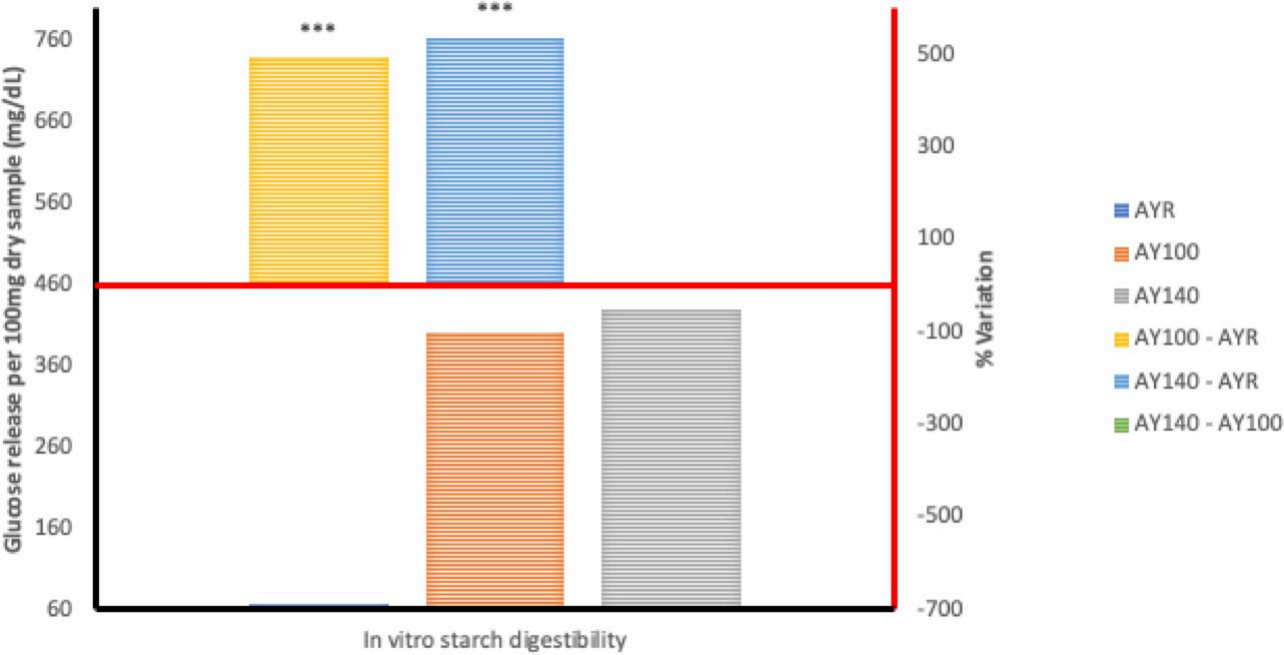
Starch digestibility of HTST extruded African yam beans (*Sphenostylis stenocarpa*) at different temperatures. AYR—unextruded African yam beans (*Sphenostylis stenocarpa*); AY100—African yam beans (*Sphenostylis stenocarpa*) extruded at 100°C; AY140—African yam beans (*Sphenostylis stenocarpa*) extruded at 140°C.

**Fig 8:**
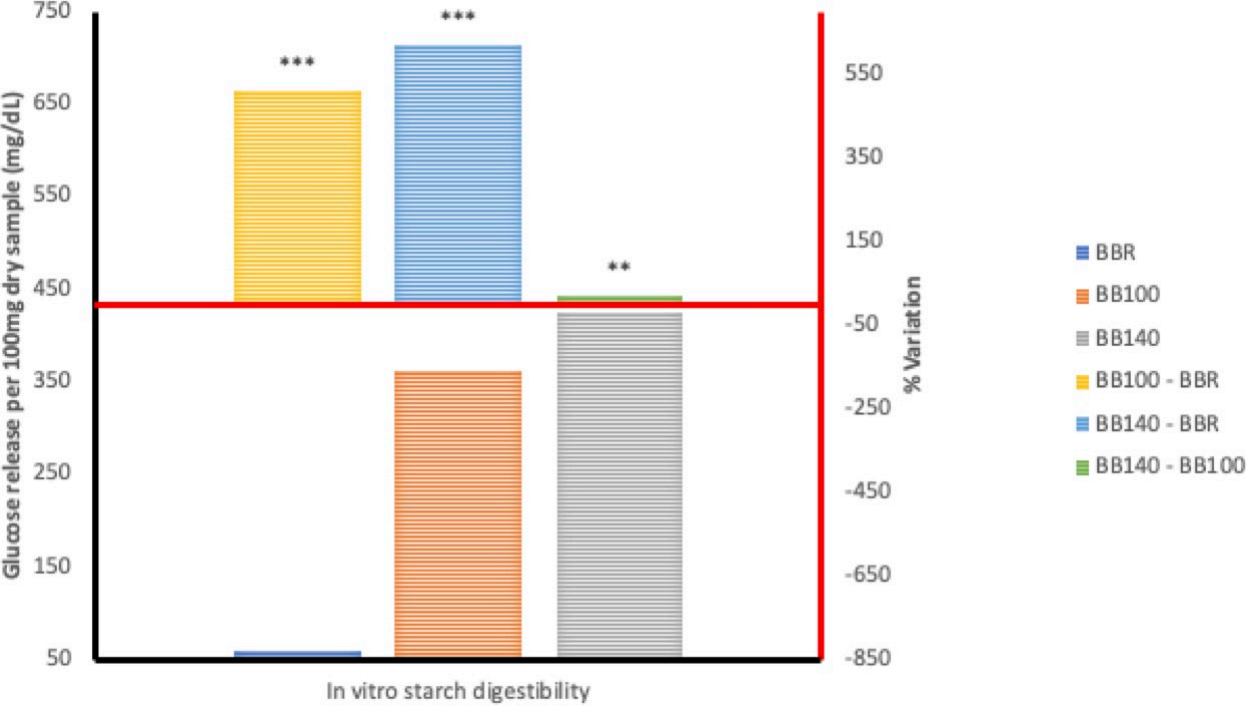
Starch digestibility of HTST extruded Bambara groundnut (*Vigna subterranean*) at different temperatures. BBR—unextruded Bambara groundnut (*Vigna subterranean*); BB100—Bambara groundnut (*Vigna subterranean*) extruded at 100°C; BB140—Bambara groundnut (*Vigna subterranean*) extruded at 140°C.

**Fig 9:**
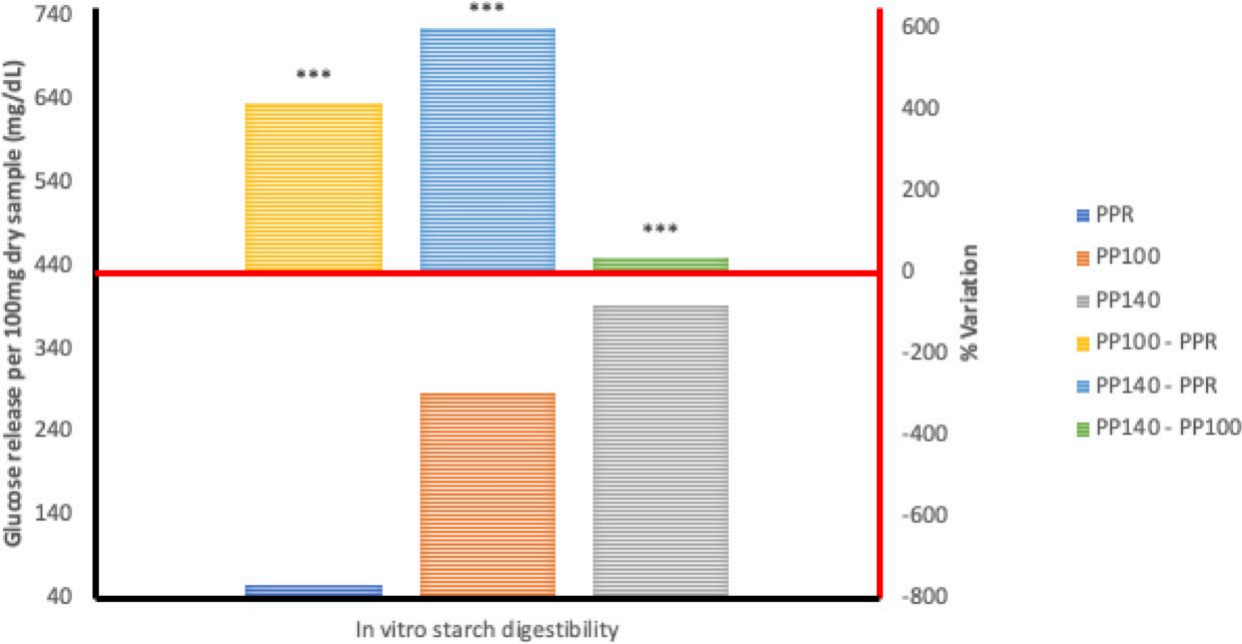
Starch digestibility of HTST extruded Pigeon pea (*Cajanus cajan*) at different temperatures. PPR—unextruded Pigeon pea (*Cajanus cajan*); PP100—Pigeon pea (*Cajanus cajan*) extruded at 100°C; PP140—Pigeon pea (*Cajanus cajan*) extruded at 140°C.

Generally, the HTST extrusion process modified starch digestion kinetics of the different grain legumes to varying degrees, hence the distinct differences in starch digestion patterns reflected in the digestograms. A two-fold increase in D_o_, gastric digestion of starch (simulated mouth and stomach phases) in response to extrusion at 100°C was recorded for *Sphenostylis stenocarpa* and *Vigna subterranean* but not *Cajanus cajan* flours. Starch rate constant (K) was unaffected by extrusion cooking at either temperatures, with no difference observed due to temperatures (100°C *vs* 140°C) except for *Cajanus cajan* flours. Area under the curve (AUC), hydrolysis index (HI) and predicted glycemic index (pGI) were significantly modified by extrusion cooking at 100°C and 140°C (p = 0.001) in *Sphenostylis stenocarpa*, *Cajanus cajan* and *Vigna subterranean* flours. However, while a significant effect of increased extrusion temperature (100°C vs 140°C) on AUC, HI and pGI was recorded in *Cajanus cajan* flours, no significant (p > 0.05) response to increased temperature was observed on these indices in *Sphenostylis stenocarpa* flours. No effects of extrusion temperatures (100°C vs 140°C) on AUC and pGI was observed in *Vigna subterranean* flours. Starch digestibility of *Sphenostylis stenocarpa*, *Cajanus cajan* and *Vigna subterranean* flours *in vitro* was significantly increased by extrusion cooking at 100°C and 140°C (p = 0.001). Increased extrusion temperature (100°C vs 140°C) also significantly increased *in vitro* starch digestibility in *Cajanus cajan* and *Vigna subterranean* flours but not in *Sphenostylis stenocarpa* flours (Figs 7–9).

## Discussion

Hydration properties have been successfully used in the screening of feed materials for different botanical origin/source [45,46] and varietal [47–49] differences, effects of processing techniques as well as effects of extrusion variables such as processing temperature, moisture [38], and mechanical stress. Increased water absorption potential of legume induced by extrusion have been reported for common bean (*Phaseolus vulgaris*), pinto and navy beans and legume pasta of faba beans, lentils and black gram beans extruded at 80–180°C [31,50–52] and range from 30.5–165% in these grain legumes. Similar extrusion-induced decrease in water solubility of extruded legumes as recorded for extrudates of *Vigna subterranean* flour in the current study were also reported for HTC *Phaseolus vulgaris* extrudates as temperature increased from 100–180°C [53,54]. Extrusion-induced decrease in water solubility of extruded legumes is perceived to be due to decreased protein solubility due to protein denaturation, which ultimately reflects on overall water solubility of the extruded legume product [54,55]. Howbeit in other studies, extrusion cooking seemed to increase water solubility of navy and pinto bean flours by 81.6% and 109.5% respectively [52], and common beans by 15.88–17.91% [31]. Extrusion-induced modifications to hydration properties of legumes have been attributed to dextrinization of the starch granule which is governed by starch and fibre content, swelling power and strength of protein network [50] of the grain legume.

The RVA pasting parameters provide a relative measure of extent of gelatinization, disintegration, swelling and gelling of the starch component of the feed material attributable to extrusion and extrusion cooking temperatures. Similar decrease in RVA peak and final viscosity was reported for cowpeas in response to extrusion at 124–160°C [56] and navy and pinto beans extruded at 85°C, with a decrease in pasting temperature and no significant effect on breakdown viscosity [52]. Setback viscosity which implies degree of re-association, retrogradation and reordering of cooked starch after cooling was also significantly decreased in cowpea extrudates [56] at different extrusion temperatures as in the current study, indicative of little or no deterioration of product starch quality due to extrusion cooking at both temperatures. The combination of heat, mechanical shear and pressure, breakdown intermolecular hydrogen bonding within the starch structure causing “melting” in extruded legume starches at lower temperatures than native legume starches [34,57]. Higher protein content of grain legumes and their availability to form protein-starch matrices are considered responsible for the absence or unresponsiveness of breakdown viscosity in grain legumes [52]. Starch digestibility *in vitro* is known to correlate well with *in vivo* digestibility [58], and extrusion-induced increase in starch digestibility reported to increase with temperature and to varied degrees in different grain legumes [28,30]. A study by Masoero et al., [59] on the effect of extrusion on *in vitro* starch digestibility of peas, faba beans and lupins showed a higher extrusion-induced increase (4.5, 11.4 and 3.4 folds, respectively) in α-amylase susceptibility of the grain legumes at the 30mins time point compared to 120 mins time point (3.4, 7.47 and 1.8 folds, respectively). This is indicative of an increase in rapidly digestible starch fractions due to extrusion cooking conditions, and similar to the marked “steepness” in the digestograms of *Sphenostylis stenocarpa*, *Cajanus cajan*, and *Vigna subterranean* extrudates compared to their raw flours in the current study.

### HTST extrusion of grain legumes: Nutritional and physiological implications for monogastric farm animals

Besides being protein sources in feeds, grain legumes contain significant complex carbohydrates; starch and NSPs, which have the potential to influence the dynamics of digestion and nutrient absorption in monogastric animals in diverse ways. Typical diets of monogastric farm animals consist of about 50% carbohydrates [60], with starch contributing 80–90% by weight [5] and more than 50% of the apparent metabolizable energy [36,61,62]. Based on *in vitro* measurements, nutritional research classifies starch into three fractions based on their digestion rate within the small intestine; rapidly digesting starch (RDS), slowly digestible starch (SDS) and resistant starch (RS). Typically, raw grain legumes contain 10.02–25.26% RDS, 21.84–39.64% SDS and 43.57–67.61% RS [63], which makes them poorly digested in the monogastric stomach. However, extrusion cooking has been shown to increases the proportion of RDS to SDS and RS in grain legumes [36] as a result of structural changes that occur in starch during extrusion which include gelatinization, melting and fragmentation [64]. This allows for increased starch digestibility *in vivo*, higher glycemic index and reduced gas production which is often observed with consumption of diets high in RS and “flatulence-causing” oligosaccharides such as raffinose and stachyose [65].

Although individual NSPs possess distinctly different chemical structures and functions, they are indigestible in the stomach and small intestinal segments of the gastrointestinal tract of monogastric animals due to an absence of fibrolytic enzymes that facilitate their digestion [60]. These NSPs are thus able to confer their unique hydration and rheological characteristics on the digesta, influencing the dynamics of digestion and nutrient absorption, and ultimately productivity of the animals. For instance, arabinoxylans and β-glucans are water soluble non-starch polysaccharides of significant importance in the nutrition of monogastric animals because they are present in viscous cereals, significantly wheat, rye, oat and barley [66–68]. When viscous cereals are used as the primary energy sources in the diets of chickens, the hydrocolloid forming ability of the inherent arabinoxylans and β-glucans modify the rheology of the digesta within the gastrointestinal tract. The resulting viscous digesta within the gastrointestinal tract of monogastric animals fed diets high in soluble NSPs has been implicated in their reduced productivity [69] by: (a) depressing feed intake, decreasing interaction between ingesta and digestive enzymes [70], and reducing small intestinal nutrient absorption rates, (b) increasing endogenous secretions, (c) delayed transition of digesta through the gastrointestinal tract, and (d) proliferation of microorganisms in the distal small intestine of poultry and colon of pigs, with resultant gastroenteritis. Studies by Yaghobfor and Kalantar [69] observed a 36.48% and 22.64%increase in digesta viscosity, and an 8.42% and 7.89% decline in productivity (measured as feed conversion ratio) of chickens on wheat and barley-based diets respectively, compared to the control chickens on a maize-based diet. These authors also reported significant proliferation of the bacterial species; *E*. *coli* and Clostridia and decline in Bifidobacteria populations in chickens on the wheat and barley-based diets. Similar trends in response to water soluble NSPs in poultry and pigs have also been reported by other researchers [71–73]. Grain legumes on the other hand are low in water soluble NSPs [68] with their predominant NSPs being insoluble NSPs; xyloglucans and galactomannans, which do not lend to increased viscosity [70] in the gastrointestinal tract. The process of extrusion cooking further lowers the rheological properties of *Sphenostylis stenocarpa*, *Cajanus cajan*, and *Vigna subterranean* extrudates, alluding to their potential for maintaining sound gut health and productivity in monogastric farm animals.

The tendency for starch retrogradation -i.e. the re-association of disaggregated amylose and amylopectin chains of gelatinized starch [74]- in a starch paste is implied by the setback viscosity index and largely dependent on time and storage temperature [74,75]. Retrogradation is associated with adverse effects on starch digestibility in monogastric animals and sensorial properties [76]. The higher lipid content of grain legumes may provide a supply of free-lipids which could retard starch retrogradation during extrusion and/or storage by constraining the mobility of amylose [77], formation of lipid-amylose complexes and lipid-amylopectin complexes with outer branches of amylopectin [74,78]. The limited tendency of grain legumes to retrograde may be beneficial for feed quality in storage and effectively retain the increased starch digestibility attained by extrusion cooking of grain legumes. Overall, the hydration characteristics, viscoamylolytic properties and *in vitro* starch digestibility of starchy feed ingredients correlate well with starch digestibility within the gastrointestinal tract of monogastric animals [58], with documented evidence of extrusion-induced increase in starch digestibility of grain legumes available for poultry [79] and swine [36].

In summary, extrusion cooking at either 100°C or 140°C modified water retention capacity and swelling power of African yam beans (*Sphenostylis stenocarpa*), Pigeon pea (*Cajanus cajan*), and Bambara peanut (*Vigna subterranean*), but not water solubility index of *Sphenostylis stenocarpa* and *Cajanus cajan* extrudates. Extrusion cooking at either 100°C or 140°C also significantly decreased peak, trough, final and setback viscosities of extrudates, with no observed differences in viscosity indices when extrusion temperatures were compared. Starch digestibility also increased with extrusion temperature, even as rapidly digestible starch fractions were markedly increased in all grain legumes investigated. Findings from this research encourage further research into the use of “lesser known grain legumes” as food and feedstuffs, which could drive increased cultivation and improvement of these species.

### Novelty statement

This research sheds light on changes observed in physicochemical and viscoamylolytic properties, and enzyme susceptibility, digestion kinetics plus glycemic index of starches of grain legumes; *Sphenostylis stenocarpa*, *Cajanus cajan*, and *Vigna subterranean*, in response to high-temperature short-time extrusion cooking at different temperatures.

## Supporting information

S1 Table (TIF)

## References

[1] Burnham RJ, Johnson KR. South American palaeobotany and the origins of neotropical rainforests. Philos Trans R Soc B Biol Sci. 2004;359: 1595–1610. 10.1098/rstb.2004.1531 15519975 PMC1693437

[2] Wilk R, Barbosa L. Rice and Beans: A Unique Dish in a Hundred Places. Wilk R, Barbosa L, editors. London/New York: Berg; 2012.

[3] Carvalho AFU, Farias DF, da Rocha-Bezerra LCB, de Sousa NM, Cavalheiro MG, Fernandes GS, et al Preliminary assessment of the nutritional composition of underexploited wild legumes from semi-arid Caatinga and moist forest environments of northeastern Brazil. J Food Compos Anal. 2011;24: 487–493. 10.1016/j.jfca.2011.01.013

[4] USDA. National nutrent database for standard reference. 2018.

[5] Hoover R, Hughes T, Chung HJ, Liu Q. Composition, molecular structure, properties, and modification of pulse starches: A review. Food Res Int. 2010;43: 399–413. 10.1016/j.foodres.2009.09.001

[6] Sethy K, Mishra SK, Mohanty PP, Agarawal J, Meher P, Satapathy D, et al An overview of Non Starch Polysaccharide. J Anim Nutr Physiol. 2015;1: 17–22.

[7] Christl SU, Murgatroyd PR, Cummings JH, Gibson GR. Hydrogen in the Large Intestine. Gastroenterology. 1992;102: 1269–1277. 1551534

[8] Bednar GE, Patil AR, Murray SM, Grieshop CM, Merchen NR, Fahey GC. Starch and Fiber Fractions in Selected Food and Feed Ingredients Affect Their Small Intestinal Digestibility and Fermentability and Their Large Bowel Fermentability In Vitro in a Canine Model. J Nutr. 2001;131: 276–286. 10.1093/jn/131.2.276 11160546

[9] Grains & Legumes Nutrition Council. Glycemic Index (GI) of Legumes. 2020 [cited 19 Oct 2020]. Available: https://www.glnc.org.au/legumes-2/legumes-and-nutrition/glycemic-index-gi-of-legumes/

[10] Jenkins DJA, Kendall CWC, Augustin LSA, Franceschi S, Hamidi M, Marchie A. Glycemic index: overview of implications in health and disease 1–4. 2002;76: 266–273. 10.1093/ajcn/76/1.266S 12081850

[11] Augustin LSA, Kendall CWC, Jenkins DJA, Willett WC, Astrup A, Barclay AW, et al Glycemic index, glycemic load and glycemic response: An International Scientific Consensus Summit from the International Carbohydrate Quality Consortium (ICQC). Nutr Metab Cardiovasc Dis. 2015;25: 795–815. 10.1016/j.numecd.2015.05.005 26160327

[12] Foster-Powell K, Holt SH a, Brand-Miller JC. International table of gylcemic index and glycemic load values: 2002. Am J Clin Nutr. 2002;76: 5–56. 10.1093/ajcn/76.1.5 12081815

[13] Wadhawan N, Wadhawan G. Diabetes and Glycemic Index: Influence of Various Foods. Acta Sci Nutr Heal. 2019;3: 119–126.

[14] Sathya A, Siddhuraju P. Effect of processing methods on compositional evaluation of underutilized legume, Parkia roxburghii G. Don (yongchak) seeds. J Food Sci Technol. 2015;52: 6157–6169. 10.1007/s13197-015-1732-4 26396363 PMC4573156

[15] Adeleye OO, Awodiran ST, Ajayi AO, Ogunmoyela TF. Effect of high-temperature, short-time cooking conditions on *in vitro* protein digestibility, enzyme inhibitor activity and amino acid profile of selected legume grains. Heliyon 2020; 6: e05419. 33225089 10.1016/j.heliyon.2020.e05419 PMC7662876

[16] Kouakou J-MK, N’Dri DY, Kouassi NK, Amani GN. Influence of hydrothermal treatment on physicochemical characteristics of white beans seeds (*Phaseolus vulgaris*) produced in Côte d’Ivoire. J Appl Biosci. 2019;127: 12849 10.4314/jab.v127i1.9

[17] Ari MM, Ayanwale BA, Adama TZ. Evaluation of different processing methods of soya beans (Glycine max) on its nutritive value and the performance of broilers: A qualitative selection approach for extension. Int J Livest Prod. 2017;8: 113–124. 10.5897/ijlp2017.0371

[18] Offiah V, Kontogiorgos V, Falade KO. Extrusion processing of raw food materials and by-products: A review. Crit Rev Food Sci Nutr. 2019;59: 2979–2998. 10.1080/10408398.2018.1480007 29787291

[19] Gregson CM, Lee TC. Quality modification of food by extrusion processing Advances in Experimental Medicine and Biology. Springer, Boston, MA; 2004 pp. 187–200. 10.1007/978-1-4419-9090-7_13 15174581

[20] Pawar SG, Pardeshi IL, Borkar PA, Rajput MR. Optimization of Process Parameters of Microwave Puffed Sorghum Based Ready- to-Eat (RTE) Food. J Ready to Eat Food. 2014;1: 59–68.

[21] Mesquita de BC, Leonel M, Mischan MM. Effects of processing on physical properties of extruded snacks with blends of sour cassava starch and flaxseed flour. Food Sci Technol Campinas. 2013;33: 404–410. 10.1590/S0101-20612013005000073

[22] Corsato Alvarenga I, Aldrich CG. Starch characterization of commercial extruded dry pet foods. Transl Anim Sci. 2020;4: 1017–1022. 10.1093/tas/txaa018 32705018 PMC7201077

[23] Monti M, Gibson M, Loureiro BA, Sá FC, Putarov TC, Villaverde C, et al Influence of dietary fiber on macrostructure and processing traits of extruded dog foods. Anim Feed Sci Technol. 2016;220: 93–102. 10.1016/j.anifeedsci.2016.07.009

[24] Kaur M, Sandhu KS, Ahlawat RP, Sharma S. In vitro starch digestibility, pasting and textural properties of mung bean: effect of different processing methods. J Food Sci Technol. 2015;52: 1642–1648. 10.1007/s13197-013-1136-2 25745235 PMC4348271

[25] Santos IL, Schmiele M, Aguiar JPL, Steel CJ, Silva EP, Souza F das C do A. Evaluation of extruded corn breakfast cereal enriched with whole peach palm (Bactris Gasipaes, Kunth) flour. Food Sci Technol. 2020;40: 458–464. 10.1590/fst.04019

[26] Alonso dos Santos P, Caliari M, Soares Soares Júnior M, Soares Silva K, Fleury Viana L, Gonçalves Caixeta Garcia L, et al Use of agricultural by-products in extruded gluten-free breakfast cereals. Food Chem. 2019;297: 124956 10.1016/j.foodchem.2019.124956 31253306

[27] Yadav U, Singh RRB, Arora S. Evaluation of quality changes in nutritionally enriched extruded snacks during storage. J Food Sci Technol. 2018;55: 3939–3948. 10.1007/s13197-018-3319-3 30228392 PMC6133873

[28] Batista KA, Prudêncio SH, Fernandes KF. Changes in the functional properties and antinutritional factors of extruded hard-to-cook common beans (phaseolus vulgaris, l.). J Food Sci. 2010;75: 286–290. 10.1111/j.1750-3841.2010.01557.x 20492281

[29] Leonard W, Zhang P, Ying D, Fang Z. Application of extrusion technology in plant food processing byproducts: An overview. Compr Rev Food Sci Food Saf. 2020;19: 218–246. 10.1111/1541-4337.12514 33319515

[30] Rathod RP, Annapure US. Effect of extrusion process on antinutritional factors and protein and starch digestibility of lentil splits. LWT—Food Sci Technol. 2016;66: 114–123. 10.1016/j.lwt.2015.10.028

[31] Batista KA, Prudêncio SH, Fernandes KF. Changes in the functional properties and antinutritional factors of extruded hard-to-cook common beans (phaseolus vulgaris, l.). J Food Sci. 2010;75: 286–290. 10.1111/j.1750-3841.2010.01557.x 20492281

[32] Politz ML, Timpa JD, Wasserman BP. Quantitative Measurement of Extrusion-Induced Starch Fragmentation Products in Maize Flour Using Nonaqueous Automated Gel-Permeation Chromatography. Cereal Chem. 1994;71: 532–536.

[33] Orford PD, Ring SG. The functional properties of extrusion-cooked Waxy-maize starch. J Cereal Sci. 1993;18: 277–286.

[34] Mitrus M, Wójtowicz A, Kocira S, Kasprzycka A, Szparaga A, Oniszczuk T, et al Effect of extrusion-cooking conditions on the pasting properties of extruded white and red bean seeds. Int Agrophysics. 2020;34: 25–32. 10.31545/intagr/116388

[35] Goodarzi Boroojeni F, Svihus B, Graf von Reichenbach H, Zentek J. The effects of hydrothermal processing on feed hygiene, nutrient availability, intestinal microbiota and morphology in poultry—A review. Anim Feed Sci Technol. 2016;220: 187–215. 10.1016/j.anifeedsci.2016.07.010

[36] Sun T, Lærke HN, Jørgensen H, Knudsen KEB, Laerke HN, Jogensen H, et al The effect of extrusion cooking of different starch sources on the in vitro and in vivo digestibility in growing pigs. Anim Feed Sci Technol. 2006;131: 67–86. 10.1016/j.anifeedsci.2006.02.009

[37] Kiarie EG, Mills A. Role of feed processing on gut health and function in pigs and poultry: Conundrum of optimal particle size and hydrothermal regimens. Frontiers in Veterinary Science. 2019 10.3389/fvets.2019.00019 30838217 PMC6390496

[38] Al-Rabadi GJ, Torley PJ, Williams BA, Bryden WL, Gidley MJ. Effect of extrusion temperature and pre-extrusion particle size on starch digestion kinetics in barley and sorghum grain extrudates. Anim Feed Sci Technol. 2011;168: 267–279. 10.1016/j.anifeedsci.2011.04.097

[39] AACC International. Method 61‐02.01. Determination of the Pasting Properties of Rice with the Rapid Visco Analyzer. 11th ed St Paul, MN, USA: American Association of Cereal Chemists;

[40] Leach HW, McCowen L, Schoch TJ. Structure of the starch granule. 1. Swelling and solubility patterns of various starches,. Cereal Chem. 1959;36: 534–544.

[41] Sopade PA, Gidley MJ. A Rapid In-vitro Digestibility Assay Based on Glucometry for Investigating Kinetics of Starch Digestion. Starch/Starke. 2009;61: 245–255. 10.1002/star.200800102

[42] Mahasukhonthachat K, Sopade PA, Gidley MJ. Kinetics of starch digestion and functional properties of twin-screw extruded sorghum. J Cereal Sci. 2010;51: 392–401. 10.1016/j.jcs.2010.02.008

[43] Kemmer G, Keller S. Nonlinear least-squares data fitting in Excel spreadsheets. Nat Protoc. 2010;5: 267–281. 10.1038/nprot.2009.182 20134427

[44] Goñi I, Garcia-Alonso A, Saura-Calixto F. A starch hydrolysis procedure to estimate glycemic index. Nutr Res. 1997;17: 427–437. 10.1016/S0271-5317(97)00010-9

[45] Miano AC, Sabadoti VD, Pereira J da C, Augusto PED. Hydration kinetics of cereal and pulses: New data and hypothesis evaluation. J Food Process Eng. 2018;41: 1–8. 10.1111/jfpe.12617

[46] Boucheham N, Galet L, Patry S, Zidoune MN. Physicochemical and hydration properties of different cereal and legume gluten‐free powders. Food Sci Nutr. 2019;7: 3081–3092. 10.1002/fsn3.1170 31572601 PMC6766534

[47] Isa J, Oyerinde AS, Jimoh KA, Jegede AO. Modelling of Hydration Characteristics of Five Varieties of Cowpea Grains. Asian Food Sci J. 2019;8: 1–16. 10.9734/afsj/2019/v8i329991

[48] Kinyanjui PK, Njoroge DM, Makokha AO, Christiaens S, Ndaka DS, Hendrickx M. Hydration properties and texture fingerprints of easy-and hard-to-cook bean varieties. Food Sci Nutr. 2015;3: 39–47. 10.1002/fsn3.188 25650021 PMC4304561

[49] An D, Arntfield SD, Beta T, Cenkowski S. Hydration properties of different varieties of Canadian field peas (Pisum sativum) from different locations. Food Res Int. 2010;43: 520–525. 10.1016/j.foodres.2009.09.034

[50] Laleg K, Cassan D, Barron C, Prabhasankar P, Micard V. Structural, culinary, nutritional and anti-nutritional properties of high protein, gluten free, 100% legume pasta. PLoS One. 2016;11: 1–19. 10.1371/journal.pone.0160721 27603917 PMC5014310

[51] Avin D, Kim C, Maga JA. Effect of extrusion variables on the physical charcateristics of red bean (Phaseolis vulgaris) flour extrudates. J Food Process Preserv. 1992;16: 327–335. 10.1111/j.1745-4549.1992.tb00213.x

[52] Siddiq M, Kelkar S, Harte JB, Dolan KD, Nyombaire G. Functional properties of flour from low-temperature extruded navy and pinto beans (Phaseolus vulgaris L.). LWT—Food Sci Technol. 2013;50: 215–219. 10.1016/j.lwt.2012.05.024

[53] Karanja C, Njeri-Maina G, Martin-Cabrejas MA, Esteban RM, Grant G, A P, et al Extrusion-induced modifications to physical and antinutritional properties of hardto- cook beans In: Fenwick GR, Hedley C, Richards RL, Khokhar S, editors. Agri-Food Quality: An Interdisciplinary Approach. The Royal Society of Chemistry: Cambridge, U.K; 1996 pp. 279–283.

[54] Martin-Cabrejas MA, Jaime L, Karanja C, Downie AJ, Parker ML, Lopez-andreu FJ, et al Modifications to Physicochemical and Nutritional Properties of Hard-To-Cook Beans (Phaseolus vulgaris L.) by Extrusion Cooking. J Agric Food Chem. 1999;47: 1174–1182. 10.1021/jf980850m 10552434

[55] Pham CB, Rosario RRD. Studies on the development of texturized vegetable products by the extrusion process. II. Effects of extrusion variables on the available lysine, total and reducing sugars. Int J Food Sci Technol. 1984;19: 549–559. 10.1111/j.1365-2621.1984.tb01871.x

[56] Hashimoto JM, Schmiele M, Nabeshima EH, Analyzer RV, Minoru Hashimoto J, Schmiele M, et al Pasting properties of raw and extruded cowpea cotyledons flours. Brazilian J Food Technol. 2020;23: 1–11. 10.1590/1981-6723.30319

[57] Thymi S, Krokida MK, Pappa A, Marinos-Kouris & D. Melting Temperatures of Extruded Products with Texturized Proteins. Int J Food Prop. 2008;11: 1–12. 10.1080/10942910601118722

[58] Doucet FJ, White G, Wiseman J, Hill SE. Physicochemical Changes to Starch Structure During Processing of Raw Materials and Their Implications for Starch Digestibility in Newly weaned Piglets In: Garnsworthy PC, Wiseman J, editors. Recent Advances in Animal Nutrition. Nottingham: Informa UK Limited; 2006 pp. 313–330. 10.5661/recadv-06-313

[59] Masoero F, Pulimeno AM, Rossi F. Effect of extrusion, espansion and toasting on the nutritional value of peas, faba beans and lupins. Ital J Anim Sci. 2005;4: 177–189. 10.4081/ijas.2005.177

[60] Halas V, Babinszky L. Role of Dietary Polysaccharides in. 2014. 10.1201/b17121-19

[61] Aar BPJ Van Der. Getting to know starch better. Feed Mix. 2003;11: 16–18.

[62] Doucet FJ, White GA, Wulfert F, Hill SE, Wiseman J. Predicting in vivo starch digestibility coefficients in newly weaned piglets from in vitro assessment of diets using multivariate analysis. Br J Nutr. 2010;103: 1309–1318. 10.1017/S0007114509993217 20021701

[63] Bravo L. Effect of processing on the non-starch polysaccharides and in vitro starch digestibility of legumes. Food Sci Technol Int. 1999;5: 415–423.

[64] Abd El-Khalek E, Janssens GPJ. Effect of extrusion processing on starch gelatinisation and performance in poultry. Worlds Poult Sci J. 2010;66: 53–63. 10.1017/S0043933910000073

[65] Borejszo ZB, Khan KH. Reduction of Flatulence‐Causing Sugars by High Temperature Extrusion of Pinto Bean High Starch Fractions. J Food Sci. 1992;57: 771–777. 10.1111/j.1365-2621.1992.tb08093.x

[66] Wood PJ. Oat and rye β-glucan: Properties and function. Cereal Chem. 2010;87: 315–330. 10.1094/CCHEM-87-4-0315

[67] Mendis M, Leclerc E, Simsek S. Arabinoxylans, gut microbiota and immunity Carbohydrate Polymers. Elsevier Ltd; 2016 pp. 159–166. 10.1016/j.carbpol.2015.11.068 26794959

[68] Choct M. Feed non-starch polysaccharides for monogastric animals: Classification and function. Anim Prod Sci. 2015;55: 1360–1366. 10.1071/AN15276

[69] Yaghobfar A, Kalantar M. Effect of Non–Starch Polysaccharide (NSP) of Wheat and Barley Supplemented with Exogenous Enzyme Blend on Growth Performance, Gut Microbial, Pancreatic Enzyme Activities, Expression of Glucose Transporter (SGLT1) and Mucin Producer (MUC2) Genes of Broiler. Brazilian J Poult Sci. 2017;19: 629–638.

[70] Ramsden L. Grains Other Than Cereals, Non-Starch Polysaccharides 2nd ed Reference Module in Food Science. Elsevier Ltd; 2016 10.1016/b978-0-08-100596-5.00091-3

[71] Jacob JP, Pescatore AJ. Using barley in poultry diets-a review. J Appl Poult Res. 2012;21: 915–940. 10.3382/japr.2012-00557

[72] Tellez G, Latorre JD, Kuttappan VA, Kogut MH, Wolfenden A, Hernandez-Velasco X, et al Utilization of rye as energy source affects bacterial translocation, intestinal viscosity, microbiota composition, and bone mineralization in broiler chickens. Front Genet. 2014;5: 1–7. 10.3389/fgene.2014.00001 25309584 PMC4174888

[73] Willamil J, Badiola I, Devillard E, Geraert PA, Torrallardona D. Wheat-barley-rye-or corn-fed growing pigs respond differently to dietary supplementation with a carbohydrase complex. J Anim Sci. 2012;90: 824–832. 10.2527/jas.2010-3766 22345107

[74] Wang SS, Li C, Copeland L, Niu Q, Wang SS. Starch Retrogradation: A Comprehensive Review. Compr Rev Food Sci Food Saf. 2015;14: 568–585. 10.1111/1541-4337.12143

[75] Mavromichalis I. How gelatinization, retrogradation affect cooked cereals—Feed Strategy In: Feed Strategy [Internet]. 13 6 2016 [cited 19 Jul 2020]. Available: https://www.feedstrategy.com/pig-nutrition/how-gelatinization-retrogradation-affect-cooked-cereals/

[76] Parada J, Aguilera JM. Review: Starch matrices and the glycemic response. Food Sci Technol Int. 2011;17: 187–204. 10.1177/1082013210387712 21593288

[77] Becker A, Hill SE, Mitchell JR. Relevance of amylose-lipid complexes to the behaviour of thermally processed starches. Starch/Staerke. 2001;53: 121–130. 10.1002/1521-379X(200104)53:3/4<121::AID-STAR121>3.0.CO;2-Q

[78] Copeland L, Blazek J, Salman H, Tang MC. Food Hydrocolloids Form and functionality of starch. Food Hydrocoll. 2009;23: 1527–1534. 10.1016/j.foodhyd.2008.09.016

[79] Nalle CL, Ravindran G, Ravindran V. Extrusion of Peas (Pisum sativum L.): Effects on the Apparent Metabolisable Energy and Ileal Nutrient Digestibility of Broilers. Am J Anim Vet Sci. 2011;6: 25–30.

