# Supplementary figures and images for: Influence of extrusion cooking on physicochemical properties and starch digestion kinetics of Sphenostylis stenocarpa, Cajanus cajan, and Vigna subterranean grains
Source: PLoS One. 2020 Dec 1;15(12):e0242697. doi: 10.1371/journal.pone.0242697 (PMC7707511; doi:10.1371/journal.pone.0242697)

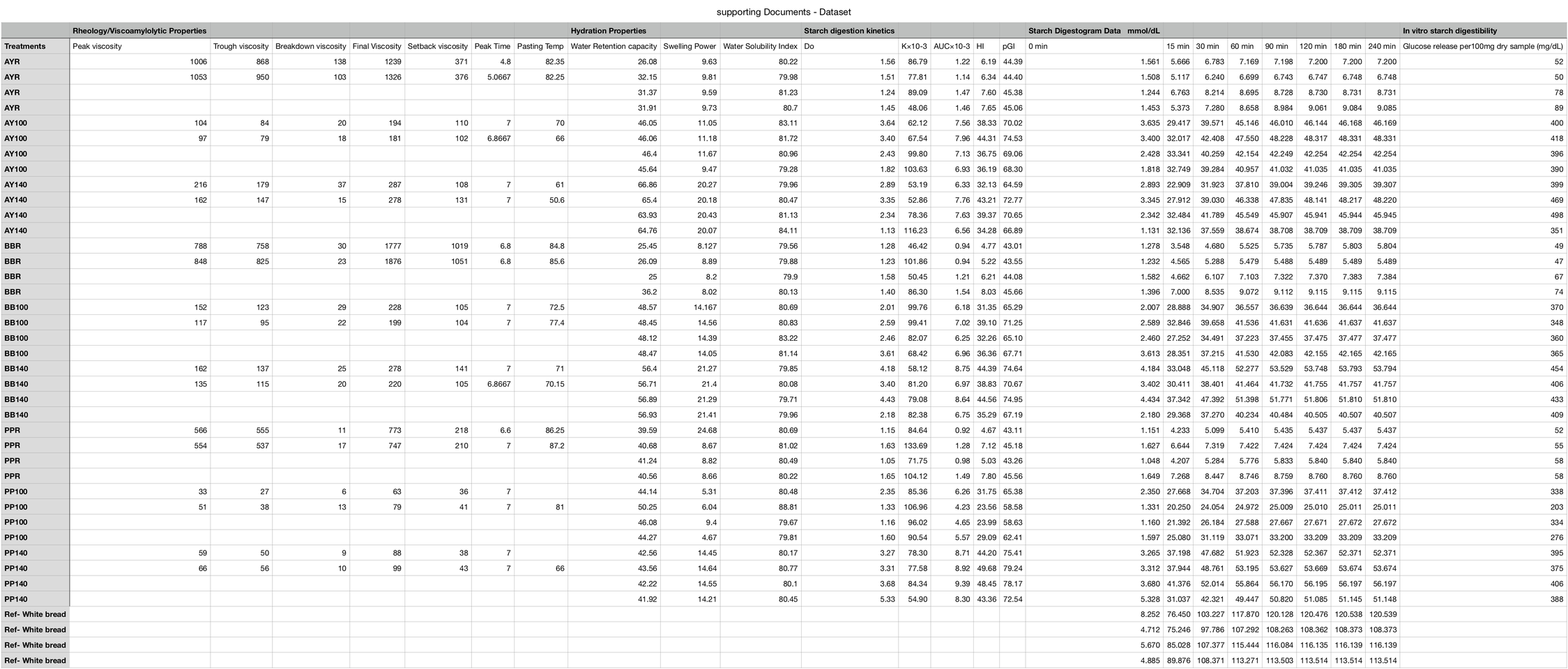

Supplement: S1 Table — (TIF) [file pone.0242697.s001.tif]
